# HflXr, a homolog of a ribosome-splitting factor, mediates antibiotic resistance

**DOI:** 10.1073/pnas.1810555115

**Published:** 2018-12-13

**Authors:** Mélodie Duval, Daniel Dar, Filipe Carvalho, Eduardo P. C. Rocha, Rotem Sorek, Pascale Cossart

**Affiliations:** ^a^Département de Biologie Cellulaire et Infection, Unité des Interactions Bactéries-Cellules, Institut Pasteur, F-75015 Paris, France;; ^b^INSERM, U604, F-75015 Paris, France;; ^c^Institut National de la Recherche Agronomique (INRA), Unité sous-contrat 2020, F-75015 Paris, France;; ^d^Department of Molecular Genetics, Weizmann Institute of Science, 76100 Rehovot, Israel;; ^e^Microbial Evolutionary Genomics, Institut Pasteur, 75015, France;; ^f^CNRS, UMR3525, 75015 Paris, France

**Keywords:** HflX, *Listeria monocytogenes*, ribosome splitting, molecular evolution, riboregulation

## Abstract

To overcome the action of antibiotics, bacteria have evolved a variety of different strategies, such as drug modification, target mutation, and efflux pumps. Recently, we performed a genome-wide analysis of *Listeria monocytogenes* gene expression after growth in the presence of antibiotics, identifying genes that are up-regulated upon antibiotic treatment. One of them, *lmo0762*, is a homolog of *hflX*, which encodes a heat shock protein that rescues stalled ribosomes by separating their two subunits. To our knowledge, ribosome splitting has never been described as an antibiotic resistance mechanism. We thus investigated the role of *lmo0762* in antibiotic resistance. First, we demonstrated that *lmo0762* is an antibiotic resistance gene that confers protection against lincomycin and erythromycin, and that we renamed *hflXr* (*hflX* resistance). We show that *hflXr* expression is regulated by a transcription attenuation mechanism relying on the presence of alternative RNA structures and a small ORF encoding a 14 amino acid peptide containing the RLR motif, characteristic of macrolide resistance genes. We also provide evidence that HflXr is involved in ribosome recycling in presence of antibiotics. Interestingly, *L. monocytogenes* possesses another copy of *hflX*, *lmo1296*, that is not involved in antibiotic resistance. Phylogenetic analysis shows several events of *hflXr* duplication in prokaryotes and widespread presence of *hflXr* in Firmicutes. Overall, this study reveals the *Listeria hflXr* as the founding member of a family of antibiotic resistance genes. The resistance conferred by this gene is probably of importance in the environment and within microbial communities.

## Significance

Antibiotics have been widely used to treat bacterial infections and are also found in the environment. Bacteria have evolved various resistance mechanisms, allowing them to overcome antibiotic exposure and raising important health issues. Here, we report a bacterial antibiotic resistance mechanism, based on ribosome splitting and recycling, ensuring efficient translation even in presence of lincomycin and erythromycin, two antibiotics that block protein synthesis. This mechanism is mediated by a HflX-like protein, encoded by *lmo0762* in *Listeria monocytogenes*, whose expression is tightly regulated by a transcriptional attenuation mechanism. This gene increases bacterial fitness in the environment. Our results raise the possibility that other antibiotic-induced resistance mechanisms remain to be discovered.

To treat bacterial infections, the use of bacteriostatic or bactericidal antibiotics remains the gold standard. These molecules act at many levels of the bacterial metabolism to prevent replication or to promote death. Beside their use as therapeutics, antibiotics are also found in the environment, since microorganisms use them to outcompete and survive within microbial communities, and many antibiotic-producing organisms such as *Streptomyces* spp. live in the soil (1).

To overcome the action of antibiotics, bacteria have evolved different resistance strategies. Resistance can be intrinsic, i.e., a natural property of the bacterium, such as the presence of an outer membrane in Gram-negative bacteria, that protects peptidoglycan from vancomycin (2); or acquired, e.g., gain of plasmid-mediated antibiotic resistance genes. The main resistance mechanisms can be classified in three major families: those that prevent the drugs from entering the cell or that actively pump them out, those that inactivate the antibiotic, and those that modify the target so that it cannot be recognized by the antibiotic (3). The genes involved in these mechanisms are often induced in presence of antibiotics, using various regulatory systems (4). One of these, called “attenuation,” relies on the presence of a 5′ regulatory region that folds into alternative RNA structures controlling either transcription or translation of the resistance gene.

*Listeria monocytogenes* is a food-borne pathogen responsible for listeriosis, a rare but lethal disease, that affects immunocompromised individuals, as well as pregnant women and elderly people (5). Although listeriosis can be efficiently treated with ampicillin and gentamicin, resistance to various antibiotics, including lincosamides, gentamicin, ampicillin, streptomycin, erythromycin, kanamycin, and rifampicin (6–12) has been reported in both food and clinical isolates.

In a previous study (13), we used a method “term-seq” to map the 3′ ends of all RNAs in bacteria grown under various conditions, including in the presence or absence of antibiotics. We discovered a previously unknown antibiotic resistance gene in *L. monocytogenes EGDe* laboratory strain, *lmo0919*, which is induced in the presence of lincomycin, an antibiotic that blocks translation by binding to the 70S ribosome in the peptidyl-transfer center, thereby preventing the transpeptidylation step (14). Another gene, *lmo0762*, is also induced in the presence of lincomycin. This gene is located downstream of rli80, a small RNA that contains a putative ORF encoding a 14-aa peptide. By sequence comparison, we found that the protein encoded by *lmo0762* is homologous to *Escherichia coli* and *Staphylococcus aureus* HflX, GTPase proteins that bind to 70S ribosomes (15–18) and recycle blocked ribosomes during heat shock in a GTP-dependent manner (15, 19, 20). Moreover, HflX was recently described as a RNA helicase that modulates rRNA conformation of heat-damaged 50S subunits in *E. coli* (21), and which is also able to split 100S disomes in *S. aureus* (20). The specific activation of *lmo0762* by sublethal doses of antibiotics suggests that this gene is involved in antibiotic resistance. However, to our knowledge, such a mechanism of ribosome splitting has not been reported in the context of antibiotic resistance.

In this study, we assessed the role of *lmo0762* in antibiotic resistance. We showed that *lmo0762* confers resistance to lincomycin and erythromycin, two antibiotics that block translation. Thus, we renamed it *hflXr*, for *hflX* resistance. We analyzed its expression regulation, and showed that an attenuation mechanism leading to premature transcription termination controls *hflXr* expression. This attenuation mechanism relies on the 14-aa ORF encoded by rli80, which strikingly contains the arginine–leucine–arginine (RLR) motif, a signature feature for macrolide resistance gene leader peptides (22). Moreover, by analyzing the polysome profiles of bacteria grown in the presence of erythromycin, we provide data suggesting that HflXr promotes recycling of blocked 70S ribosomes and thus translation. Finally, we identified another *L. monocytogenes* homolog of *hflX*, *lmo1296*, which is not involved in antibiotic resistance. Phylogenetic analysis revealed that duplication occurred several times, independently, in several major clades of prokaryotes, and that many Firmicutes possess a *hflX* copy closely related to *hlfXr*. Overall, these data suggest that this antibiotic resistance mechanism, as with other resistance mechanisms, is probably of importance for survival in the environment and within microbial communities.

## Results

### *lmo0762* Is an Antibiotic Resistance Gene.

**Fig. 1.**
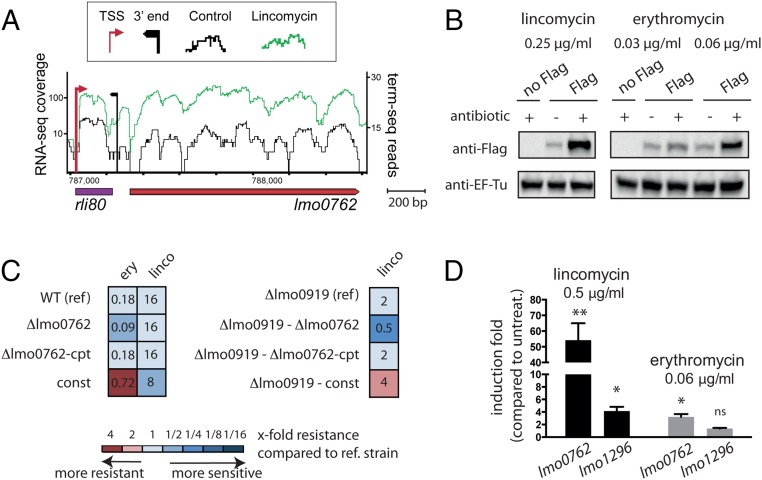
*lmo0762* is an antibiotic resistance gene. (*A*) RNA-seq profile of *rli80-lmo0762* locus obtained from *L. monocytogenes* grown in absence (black) or in presence of lincomycin (0.5 µg/mL, green). (*B*) Western blot analysis performed on WT (no Flag) and a strain expressing Lmo0762 with a Flag at the C terminus (Flag). EF-Tu was used as a loading control. (*C*) Schematic representation of MIC assay. The colors indicate that the tested strain is more sensitive (blue) or more resistant (red) to the antibiotic compared with the reference (ref) strain. The MIC values are indicated in the boxes after 48-h incubation (in μg/mL). (*D*) Induction of *lmo0762* and *lmo1296* upon antibiotic treatment calculated by qPCR in comparison with the endogenous level before addition of the antibiotic. Data are represented as mean ± SEM. We used a one-way ANOVA on ΔCt values for statistics, using biological replicates as pairing factors. *P < 0.05, **P < 0.01; ns, nonsignificant.

In a previous study, we analyzed the expression profile of all *Listeria* genes in the presence of subinhibitory concentrations of lincomycin and discovered that *lmo0762* is induced (ref. 13 and Fig. 1*A*; data are publicly available in the European Nucleotide Database, accession no. PRJEB25942). This gene is located downstream of a small RNA, rli80, and while rli80 appears to be transcribed in both the absence (black) or the presence (green) of lincomycin, *lmo0762* is primarily transcribed in the presence of the antibiotic. To verify *lmo0762* induction by lincomycin at the protein level, we created a strain in which a Flag tag was introduced at the C terminus of the Lmo0762 protein. We extracted total proteins from this strain after growth in the absence or presence of subinhibitory concentration of the antibiotic, and performed a Western blot using an anti-Flag antibody (Fig. 1*B*, *Left*). The results confirmed that Lmo0762 levels are significantly increased in presence of lincomycin. This up-regulation prompted us to investigate whether *lmo0762* plays a role in antibiotic resistance.

To test this hypothesis, we constructed different strains: a mutant strain with a deleted *rli80-lmo0762* region (*Δlmo0762*); a complemented strain, where the *rli80-lmo0762* region, under the control of its native promoter, was reintroduced at a different locus of the *Δlmo0762* strain (*Δlmo0762-cpt*); and a strain constitutively expressing Lmo0762, independently of the presence of antibiotics, due to a mutation in the upstream regulatory region that we describe below (*SI Appendix*, Fig. S1, *const*). We confirmed by qRT-PCR that *lmo0762* is induced by lincomycin at similar levels in the wild type (WT) and *Δlmo0762-cpt* strains (*SI Appendix*, Fig. S2). We then performed a minimum inhibitory concentration (MIC) assay on these strains using various antibiotics (Fig. 1*C*, *Left* and *SI Appendix*, Fig. S3), and we observed that the *Δlmo0762* strain is twofold more sensitive to erythromycin than the WT and *Δlmo0762-cpt* strains, a result consistent with effects observed with several macrolide resistance determinants (23). In contrast, the constitutively overexpressing *const* strain is fourfold more resistant to erythromycin than the WT. We confirmed by Western blot that Lmo0762 levels also increase in presence of erythromycin (Fig. 1*B*, *Right*), even though induction at the transcription level is lower than in presence of lincomycin (Fig. 1*D*). However, while *lmo0762* expression is induced by lincomycin exposure, the *Δlmo0762* strain did not show increased sensitivity to lincomycin, and Lmo0762 overexpression resulted in increased sensitivity to lincomycin, rather than the expected resistance. Given that we previously identified another gene, *lmo0919*, as a lincosamide resistance gene (13), we hypothesized that *lmo0919* may be masking the effect of the *lmo0762* deletion. Thus, we constructed a double mutant strain, *Δlmo0919Δlmo0762*, that we complemented by reintroducing the locus *rli80-lmo0762* elsewhere in the genome, as previously described (*Δlmo0919Δlmo0762-cpt*), and we also introduced the mutation that led to *lmo0762* constitutive overexpression (*Δlmo0919-const*). We used these four strains to perform MIC assays (Fig. 1*C*, *Right* and *SI Appendix*, Fig. S3), and strikingly, we found that the double mutant is fourfold more sensitive to lincomycin compared with the *Δlmo0919* strain, and that resistance is restored in the complemented strain. Moreover, overexpression of Lmo0762 (*Δlmo0919-const*) renders the strain more resistant to lincomycin than the *Δlmo0919* strain. Overall, we conclude that *lmo0762* is an antibiotic resistance gene, conferring protection against lincomycin and erythromycin.

### Lmo0762 Is a Homolog of the Bacterial Ribosome-Splitting Protein HflX.

To explore the mode of action of Lmo0762, we searched for homologs in other species using BLASTp and HMM protein profiles. The top hits were homologs of HflX in various bacteria, including *S. aureus*, *Bacillus cereus*, and *E. coli* (*SI Appendix*, Table S1). HflX has been described as a heat-shock stress-response GTPase that can split and recycle ribosomes that have become immobilized due to heat stress (15, 19, 20). We thus renamed *lmo0762 hflXr*, for *hflX* resistance. Surprisingly, we discovered another homolog of *hflX* in *L. monocytogenes*, *lmo1296* (*SI Appendix*, Table S1). The two proteins encoded by *hflXr* and *lmo1296* contain a predicted GTP-binding domain and a 50S ribosome-binding domain (*SI Appendix*, Table S2), like all other 8,527 homologs of this family that were found within 8,113 genomes.

**Fig. 2.**
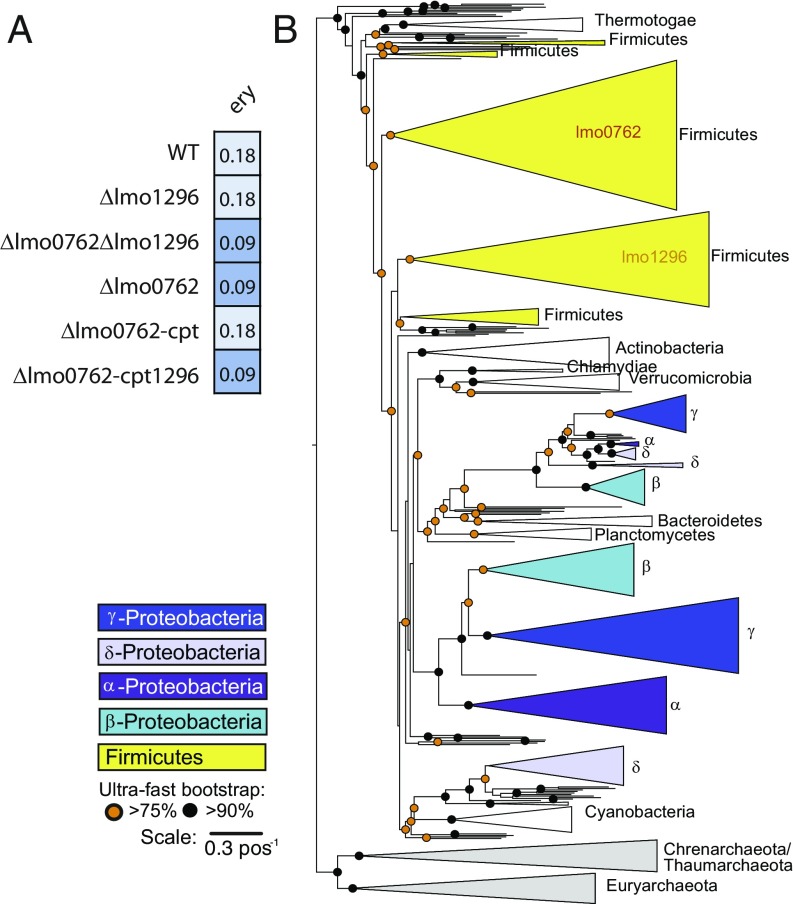
Phylogeny of HflX homologs. (*A*) Schematic representation of MIC assays, similar to Fig. 1*C*. (*B*) Schematic representation of the phylogenetic tree of *hflX* homologs among prokaryotes. A detailed tree is available in *SI Appendix*, Figs. S4 and S5.

To decipher whether *lmo1296* also participates in antibiotic resistance, we first analyzed its induction upon antibiotic exposure. qRT-PCR analysis of RNAs extracted from bacteria grown in presence or absence of antibiotics showed weak or no induction of *lmo1296* upon antibiotic exposure in comparison with *lmo0762* (Fig. 1*D*). We then performed MIC assay on a strain lacking *lmo1296* (*Δlmo1296*), which showed no increased susceptibility to erythromycin, or any other antibiotics tested, compared with WT strain (Fig. 2*A* and *SI Appendix*, Fig. S3). We also created the *hflXr-lmo1296* double deletion strain (*Δlmo0762-Δlmo1296*), which showed no further susceptibility compared with the *Δlmo0762* strain. Finally, we reintroduced the *lmo1296* gene, under the control of rli80, in the *Δlmo0762* mutant (*SI Appendix*, Fig. S2*A*, *Δlmo0762-cpt1296*). We confirmed by qRT-PCR that in this strain, *lmo1296* is induced by the antibiotic at a level similar to that of *hflXr* in the WT or *Δlmo0762-cpt* strains due to rli80 regulation (*SI Appendix*, Fig. S2*B*), and we tested the strain in a MIC assay. We observed that unlike *Δlmo0762-cpt*, the *Δlmo0762-cpt1296* strain remains sensitive to erythromycin (Fig. 2*A* and *SI Appendix*, Fig. S3), thus indicating that *lmo1296* is not involved in antibiotic resistance.

To unravel the evolutionary history of *hflXr* and its homologs, we reconstructed the phylogeny of *hflX* genes among prokaryotes (Fig. 2*B* and *SI Appendix*, Fig. S4). Strikingly, the phylogenetic tree shows that *lmo1296* and *hflXr* are well separated in two large clades, both containing almost only Firmicutes. This duplication event is probably old, since it is shared by many bacteria from the clade, and the trees of the two subfamilies closely recapitulate the tree of Firmicutes (*SI Appendix*, Fig. S5). Interestingly, other phyla also harbor a duplication in the *hflX* genes, e.g., α-, β-, γ-, and δ-proteobacteria and Archaea. These events occurred independently from the one of Firmicutes, highlighting the importance of *hflX* gene duplication. The analysis of 163 pangenomes from bacteria revealed that the genes of this family, when present, are in the core genome (98% in >90% of the strains, *SI Appendix*, Fig. S6). Finally, the analysis of genetic neighborhood of the two subfamilies showed much higher conservation for *lmo1296* than for *hflXr* (Datasets S1 and S2). Based on these elements, it is tempting to speculate that the ancient duplication of *hflX* led to two proteins with specialized functions in Firmicutes, explaining why duplicates cooccur, one of which being involved in antibiotic resistance (*hflXr*). If correct, this means that other genes of this subfamily may provide antibiotic resistance. Such genes were found in important pathogens, such as *B. cereus*, *Bacillus anthracis*, and *Clostridium difficile*.

### HflXr Recycles Ribosomes upon Antibiotic Exposure.

Ribosome stalling is a phenomenon induced by heat shock that results in translation arrest. Some antibiotics can also impair translation, and resistance proteins that remove the antibiotic from the stalled ribosome have previously been described (tetO, tetM, and ABC-F transporters) (24–26). However, a mechanism by which the ribosome is split and recycled has not been described in the context of antibiotic resistance and could constitute another class of antibiotic resistance factors.

**Fig. 3.**
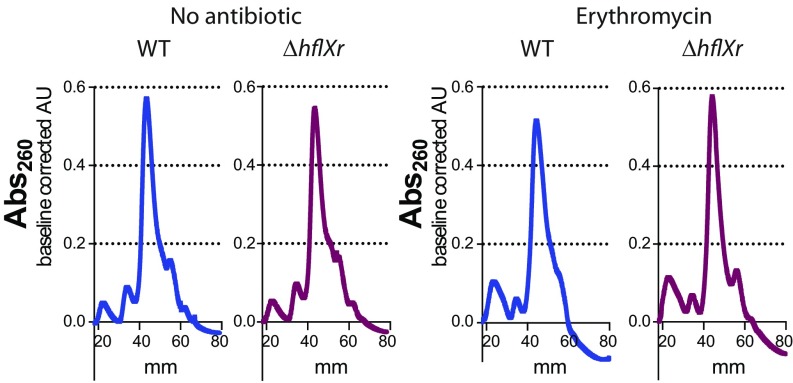
Lmo0762 is involved in recycling of antibiotic-stalled 70S ribosomes. Wild-type (blue) and *Δlmo0762* (purple) bacteria were grown in BHI medium until exponential phase and erythromycin (0.18 µg/mL) was added or not for 1 h. Chloramphenicol was then added to the culture (2-min exposure at 5 mM) to stabilize the polysomes, and bacteria were pelleted and flash frozen. The cellular content was extracted, and equal amount of lysate (A_260_) was loaded on a 5–50% sucrose gradient. After ultracentrifugation, the samples were collected from top (0 mm) to bottom (80 mm) of the tubes and the absorbance at 260 nm was monitored using a UV lamp. The baseline was corrected and the results were normalized based on the area under the curve.

To assess if HflXr promotes antibiotic resistance via ribosome splitting and recycling, we analyzed the polysome profiles in WT and *Δlmo0762* bacteria grown in the presence or absence of antibiotic. Given that *lmo0919* may mask the effect of *hflXr* in the presence of lincomycin, we used erythromycin in this experiment. The results show a greater quantity of 70S in the *Δlmo0762* compared with WT in presence of erythromycin, while this difference is not visible in absence of antibiotic (Fig. 3 and *SI Appendix*, Fig. S7). A comparable difference was previously observed in *E. coli*, in the context of heat shock (19). Such an accumulation of 70S ribosomes in absence of *hflXr* suggests that this gene participates in ribosome recycling, probably by splitting ribosomes halted upon antibiotic exposure.

### *hflXr* Transcription Is Regulated by a Ribosome-Dependent Attenuation Mechanism.

**Fig. 4.**
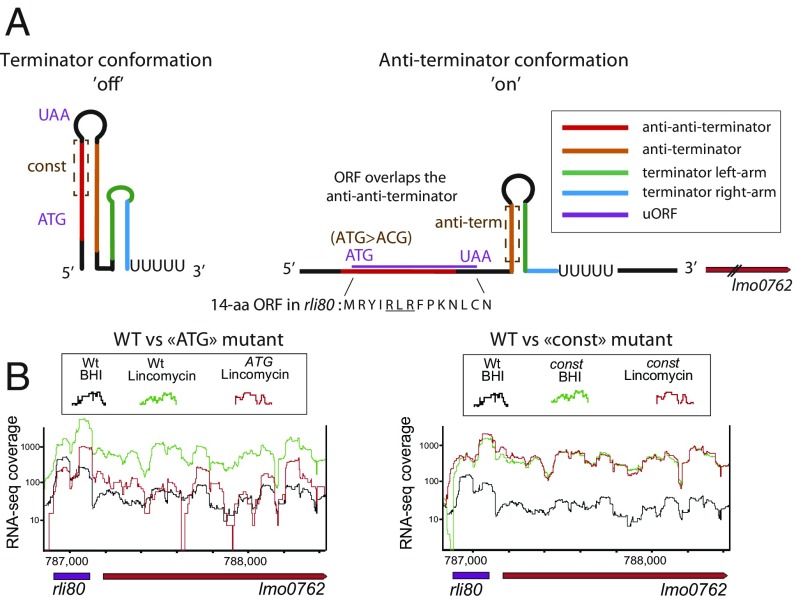
Lmo0762 expression is regulated by a transcription attenuation mechanism. (*A*) The predicted rli80 RNA structures were analyzed using the PASIFIC algorithm, and two alternative conformations were predicted, one with an intrinsic terminator (*Left*) that leads to a short transcript, and one with an anti-terminator (*Right*) that leads to a long transcript that encodes *lmo0762*. Key regulatory regions were identified (anti-anti-terminator in red, anti-terminator in orange, and terminator in green/blue) and a short ORF of 14 aa (purple) is encoded in a region that encompasses the anti-anti-terminator region. Different mutants were created where regulatory regions were removed (dashed brown squares) to decipher the regulatory mechanism. (*B*) RNA-seq profiles of WT and mutant bacteria, obtained as in Fig. 1*A*.

As shown above, *hflXr* expression is induced by lincomycin and erythromycin (Fig. 1 *A* and *D*). Moreover, *hflXr* is located downstream of rli80, a constitutively transcribed small RNA that we hypothesized to act as a regulatory switch (Fig. 1*A*). Using the term-seq data for *L. monocytogenes* grown in the absence of antibiotics (13), we found an accumulation of 3′ reads immediately downstream of the rli80 riboregulator, suggesting a regulatory mechanism relying on premature termination (4) (Fig. 1*A*, black arrow). A hallmark of such riboregulators is the ability to display mutually exclusive RNA folding patterns that either stabilize or destabilize the intrinsic transcriptional terminator, turning the downstream gene transcription “off” or “on,” respectively (4, 27, 28), in a process named “attenuation.” We thus searched for such alternative RNA structures in the rli80 sequence, using the PASIFIC algorithm (29). We found that rli80 can indeed fold into either a terminator structure, that would lead to premature termination and to an accumulation of short RNAs, or alternatively into a structure acting as an anti-terminator, which prevents the formation of the terminator, allowing the synthesis of full-length *hflXr* mRNA (Fig. 4*A* and *SI Appendix*, Fig. S8). In addition, we found that rli80 contains an ORF encoding a 14-aa peptide, which harbors an RLR motif (Fig. 4*A*), a hallmark signature found in small ORFs of other macrolide sensing attenuators (*SI Appendix*, Table S3). The RLR motif controls translation arrest by disturbing the transpeptidation step due to the amino acid geometry (22), thus promoting the expression of the downstream macrolide resistance gene (30, 31).

Based on this model as well as on the induction by antibiotics, we hypothesized that *hlfXr* transcription is controlled either by direct binding of the antibiotic to the mRNA (riboswitch) or by a ribosome-mediated attenuation mechanism due to ribosomal stalling on the rli80 ORF. To discriminate between these two possibilities, we performed RNA-seq to measure the lincomycin-dependent induction of *hflXr* in a strain expressing the 23S rRNA methyltransferase ErmC which renders ribosomes insensitive to lincomycin (32). The results show that in ErmC-expressing bacteria, *hflXr* expression was no longer activated in response to the antibiotic (*SI Appendix*, Fig. S9*A*), suggesting that the riboregulation depends on stalled ribosomes rather than by direct binding of the antibiotic to the RNA. We further tested this hypothesis by validating that the small ORF is translated in vivo by generating a strain with a translational GFP fusion to the C terminus of the small peptide (*SI Appendix*, Fig. S9*B*), that showed fluorescence under the microscope. To validate the attenuation mechanism, which involves different regulatory RNA structures and the rli80 ORF, we mutated the start codon of the ORF (ATG > ACG), as well as regions controlling the formation of the terminator, named anti-terminator and anti-anti-terminator regions (Fig. 4*A*, brown dashed squares). We analyzed the RNA-seq profile of the *rli80-lmo0762* locus in these different mutant strains, in the absence and presence of lincomycin (Fig. 4*B* and *SI Appendix*, Fig. S9*C*, data available in the ENA database, accession no. PRJEB25942). In agreement with the above hypothesis, mutating the anti-terminator region or the ORF start codon, both of which are predicted to stabilize the off conformation of rli80, prevented activation of *lmo0762* expression during lincomycin exposure and led to lower mRNA abundance in qRT-PCR assay (*SI Appendix*, Fig. S10). In contrast, mutating the anti-anti-terminator region (i.e., the strain that we previously named *const*), which is predicted to maintain the on conformation of the riboregulator, led to constitutive readthrough and expression of *hflXr*, regardless of the presence of lincomycin, as well as enhanced antibiotic resistance (Fig. 1*C* and *SI Appendix*, Figs. S3 and S10). Taken together, these results show that rli80 controls the expression of *hflXr* via a ribosome-dependent transcription attenuation mechanism, such that HflXr protein expression is induced in response to ribosome stalling.

## Discussion

In this work, we describe an antibiotic mechanism of resistance to lincomycin and erythromycin in *L. monocytogenes* which is mediated by Lmo0762, an HflX homolog, that we renamed HflXr, for HflX resistance. We showed that deletion of the gene renders the bacteria more sensitive to erythromycin and lincomycin, while its overexpression renders them more resistant. The induction of the gene in the presence of antibiotics is mediated by an attenuation mechanism that involves a small ORF encoding a peptide containing a RLR motif. This tripeptide is a signature of macrolide resistance genes, since it is commonly found in small ORFs in leader regions that regulate their expression (*SI Appendix*, Table S3) (30, 31). Moreover, by analyzing the polysome profiles in bacteria grown in the presence or absence of antibiotic, we provided evidence that the proportion of 70S ribosome increases upon erythromycin treatment in a strain lacking *hflXr*, which led us to propose that the mechanism of action of HflXr is to split ribosomes, as for *E. coli* HflX. Surprisingly, in vitro experiments showed that erythromycin and lincomycin can inhibit the GTPase activity of the *E. coli* HflX (15). Given that ribosome splitting was described as a GTP-dependent mechanism (15, 19), further investigations will be required to reconcile these data with our results.

Interestingly, *L. monocytogenes* encodes another *hflX* homolog, *lmo1296*, which is not involved in antibiotic resistance. Many Firmicutes possess two copies of *hflX*, and similar independent duplications are observed in other clades of prokaryotes (Fig. 2*B*). Given that many Firmicutes possess a *hflX* gene that belongs to the subfamily of *hflXr*, it seems reasonable to think that the antibiotic resistance mechanism that we described in *Listeria* for *hflXr* could be conserved in many other bacteria. This duplication represents an example of how bacteria can employ common stress response factors as antibiotic resistance genes. In addition, our work is now strengthened by two recently published studies where functional metagenomic databases constructed from antibiotic-rich environments pointed to *hflX* from *Simkania negevensis* and *Emergencia timonensis* as a putative resistance gene (23, 33). Based on our phylogenetic analysis, *hflX* from *Eubacterium* spp., which is closely related to *E. timonensis*, belongs to the *hflXr* family. *S. negevensis* is not a Firmicute and its *hflX* belongs to subfamilies which have not been studied for antibiotic resistance. These observations reinforce the conclusions of our study and further suggest that this antibiotic resistance mechanism is likely spread in the environment. It is interesting to note that other ribosome rescue mechanisms contribute to basal antibiotic resistance, e.g., tmRNA, whose inactivation renders *E. coli* more susceptible to antibiotics, and leads to impaired protein synthesis in presence of antibiotics (34, 35). However, a distinction should be made with *hflXr*, given that the tmRNA is a general stress factor, while *hflXr* duplication, specialization, and transcription regulation favor the idea that this gene is dedicated to response to antibiotics. In addition, functional metagenomic proves that *hflX* from other species that belong to the same subfamily as *hflXr* confer resistance to macrolides when transferred to other bacteria, which, to our knowledge, is not the case for tmRNA (23, 33). However, it has been shown that the sigma factor sigR induced by antibiotics in *Streptomyces coelicolor* regulates the transcription of various ribosome-associated genes, and notably tmRNA and HflX (36). Hence, further analysis will be required to determine whether the transtranslation and HflXr may act in a synergistic manner in ribosome recycling, or whether tmRNA may compensate the lack of *hflXr* in some bacterial species. Finally, the level of resistance conferred by *hflXr* may appear weak in clinical settings according to EUCAST breakpoints, but given that homologs of *hflXr* are spread in Firmicutes, this gene is probably of importance in an environmental context and within microbial communities, conferring resistance to antibiotics that may be found in the soil, such as lincomycin and erythromycin.

**Fig. 5.**
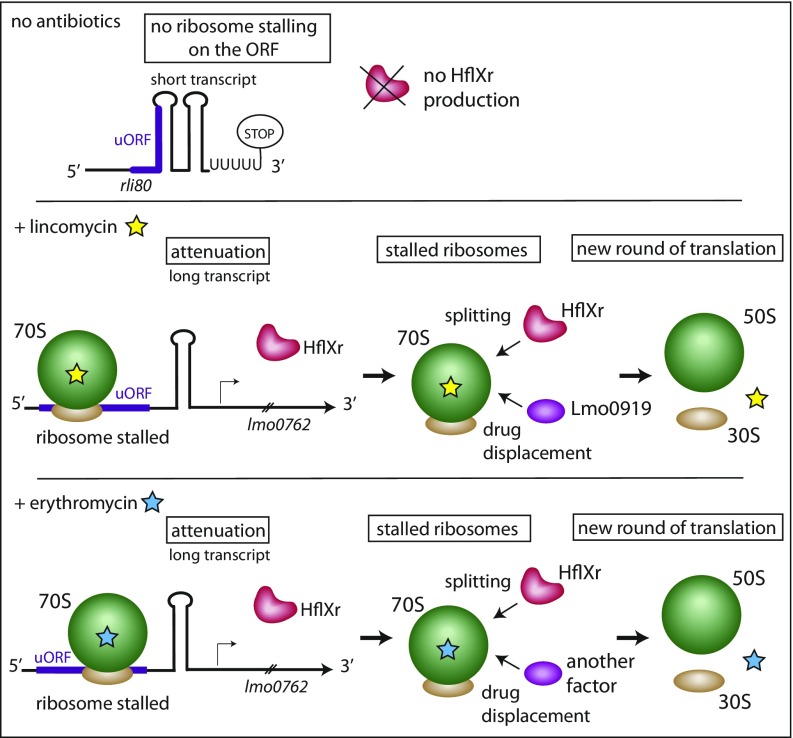
Model of the combined action of Lmo0762 and Lmo0919 to protect bacteria against lincomycin and erythromycin.

The benefit provided by *hflXr* in bacteria exposed to lincomycin and to erythromycin seems different, since it was necessary to delete *lmo0919*, another lincomycin resistance gene, to observe the effect of the *hflXr* deletion in the presence of this antibiotic, while the effect of *hflXr* could be directly visualized for erythromycin. Lincomycin belongs to the lincosamide antibiotic family, whose members bind the ribosome at the peptidyl-transfer center and inhibit peptide bond formation. Erythromycin is a macrolide antibiotic that also binds at the vicinity of the peptidyl-transfer center, in the peptide exit tunnel channel, nearby to the lincomycin target site. Both antibiotics prevent translation at early stages of elongation (32). The remaining stalled ribosomes need to be recycled to start a new round of translation. We noted that overexpression of HflXr in the Δ*lmo0919* strain (Δ*lmo0919*-*const*) partially restores resistance to lincomycin (*SI Appendix*, Fig. S3). These data led us to think that Lmo0919 and HflXr act independently to recycle the ribosome and restart translation, that Lmo0919 is of primary importance for lincomycin resistance, and that HflXr can partially rescue *lmo0919* deletion. Our careful analysis has shown that the *lmo0919* gene encodes an “ABC-F transporter” (*SI Appendix*, Fig. S11), correcting our previous assumption that it encodes an antibiotic efflux pump (13). Recent studies have shown that antibiotic resistance genes annotated as ABC-F transporters have the capability of displacing ribosome-bound antibiotics in vitro (24, 26). Interestingly, Lmo0919 is only produced in the presence of lincomycin (13) and is not involved in erythromycin resistance (*SI Appendix*, Fig. S3), while other ABC-F transporters such as MsrA confer macrolide resistance (26). Thus, we hypothesize that HflXr could act in concert with Lmo0919 in the presence of lincomycin: HflXr would split the ribosome, while Lmo0919 would displace the antibiotic, thus recycling the ribosome to restart translation. In the presence of erythromycin, HflXr could act either alone or in combination with a protein with macrolide displacement activity to rescue stalled ribosome and restart translation. This hypothesis is presented in Fig. 5.

*hflX* transcription is regulated by an attenuation mechanism that relies on the upstream regulatory RNA, rli80, which folds into alternative structures and contains a small ORF that harbors the RLR motif. This attenuation mechanism involves the pausing of antibiotic-stalled ribosomes on the ORF, which in turn prevents the formation of the terminator hairpin structure, thus permitting the transcription the full-length *hflXr* mRNA. This allows the bacteria to fine tune the expression of *hflXr* in response to two antibiotics that block translation after incorporation of a few amino acids. As a consequence, the regulation also works as a feed-forward loop, by shutting down the expression of HflXr when the antibiotic is cleared. Indeed, in the absence of drug, the ribosome does not pause on the regulatory region, and this in turn prevents *hflXr* transcription. These findings are recapitulated in our model (Fig. 5). Such an attenuation mechanism involving RNA structures and a small ORF has been found for different antibiotic resistance genes e.g., the *ermC* gene (4, 30). It is interesting to note that in this latter example, a translation attenuation modulates the availability of the ribosome-binding site of the resistance gene, while our regulation mechanism modulates the transcription of *lmo0762*.

Overall, we described here an antibiotic resistance mechanism in *L. monocytogenes*, that uses HflXr to recycle ribosomes in the presence of antibiotics. The *hflXr* gene seems widely spread across species, and it is to be expected that *hflXr* genes are employed in other bacterial species for antibiotic resistance in the environment and within bacterial communities.

## Materials and Methods

A detailed materials and methods section can be found in *SI Appendix*.

### RNA-Seq Library Preparation and Analysis.

RNA was extracted and DNase treated and chemically fragmented. Strand-specific RNA-seq libraries were prepared and sequencing was performed using the Illumina NextSEq 500. Data were deposited in the European Nucleotide Database (ENA) under accession no. PRJEB25942. Sequencing reads were mapped to the NC_003210 *L. monocytogenes* EGD-e RefSeq genome.

### Minimum Inhibitory Concentration.

Six to eight colonies were resuspended in BHI, diluted at OD_600_ = 0.001 in 96-well plates, and incubated in presence of increasing concentrations of antibiotics for 48 h at 37 °C without shaking. The MIC was determined as the lowest concentration that fully inhibits growth.

### Polysome Profiling.

In bacterial culture, erythromycin (0.18 µg/mL) was added or not (untreated condition) at OD_600_ = 0.6 and the growth was pursued for 1 h. Chloramphenicol was quickly added to every culture (2 min at 5 mM final concentration) to stabilize polysomes, and bacterial pellet was washed and flash frozen. Cellular content were extracted using a FastPrep apparatus. An equal amount (15–35K OD_260_) of cell lysates were loaded on sucrose gradient (5–50%), ultracentrifuged, and separated on a Biocomp instrument. Absorbance was read at 260 nm at a speed of 0.12 mm/s.

### Homology Analysis.

A total of 9,078 complete genomes retrieved from NCBI RefSeq representing 3,226 species of prokaryotes were analyzed. The composition of Lmo0762 and Lmo1296 in protein domains was analyzed using PFAM. We used these domains to search the database of genomes using hmmsearch.

### Phylogenic Trees.

First we reduced the redundancy of the dataset using mmseqs2 to cluster the proteins in clusters of 80% identity and 60% identity. When necessary, we added manually to the databases, the genomes that contained at least two copies of the protein to ensure that the phylogenetic reconstruction contained the two copies of each pair of duplicates. We made a phylogenetic reconstruction for each of the two datasets: we made multiple alignments with MAFFT v7.407, we collected the informative sites in multiple alignment using trimAl v1.2rev59, and made a phylogenetic reconstruction with IQtree v1.6.7. The dataset_60 reconstruction was used in the study because it had higher ultrafast bootstrap results and fewer taxa. However, the key results were common between the two reconstructions.

## Supplementary Material

Supplementary File

Supplementary File

Supplementary File

Supplementary File

Supplementary File

Supplementary File

Supplementary File
