# Supplementary material for: HflXr, a homolog of a ribosome-splitting factor, mediates antibiotic resistance
Source: Proc Natl Acad Sci U S A. 2018 Dec 13;115(52):13359–64. doi: 10.1073/pnas.1810555115 (PMC6310831; doi:10.1073/pnas.1810555115)
Supplement: Supplementary File [file pnas.1810555115.sapp.pdf]

PNAS
www.pnas.org

Supplementary Information for

HflXr mediates an antibiotic resistance mechanism

Melodie Duval[a,b,c], Daniel Dar[d], Filipe Carvalho[a,b,c], Eduardo P. C. Rocha[e,f], Rotem Sorek[d], Pascale Cossart[a,b,c*]

Pascale Cossart Email: pascale.cossart@pasteur.fr

This PDF file includes:

Supplementary Material and methods and related bibliography
Figs. S1 to S11
Tables S1 to S4

www.pnas.org/cgi/doi/10.1073/pnas.1810555115

Supplementary Material and methods

Bacterial strains, plasmids, primers and growth conditions

For standard experiments *Listeria monocytogenes* was grown overnight in Brain Heart Infusion (BHI) medium (Difco) at 37°C while shaking at 200 rpm. Overnight cultures were diluted 1/100 in fresh BHI and grown at 37°C until exponential phase (OD_{600} = 0.6-0.8). When required, 0.03 µg/ml erythromycin or 0.25 µg/ml lincomycin was added to the culture medium, as these concentration are the sub-inhibitory concentrations that we previously determined in [1]. When indicated, the concentrations were increased to 0.5 µg/ml (lincomycin) and 0.06 µg/ml (erythromycin).

For RNA-seq and qRT-PCR, samples were collected before addition of antibiotic or after 15 min exposure. For western blot, samples were collected after 1h exposure.

Listeria mutants were generated using the pMAD shuttle plasmid[2] as described previously[3, 4]. For the construction of pMAD and pAD-based plasmids, fragments obtained by PCR with EGD-e genomic DNA or synthetic DNA (Gblocks from Integrated DNA Technologies) were cloned into the EcorI/BamHI or NheI/BamHI sites of the pMAD, or SmaI/SalI sites of the pAD vector[5] derived previously from the pPL2 vector[6]. Plasmids constructs were confirmed by sequencing and transformed into *L. monocytogenes* by electroporation. For pAD, integration in the chromosome was verified by PCR using primers NC16 and PL95[6].

Strains, plasmids and primers used in this study are listed in the table S4.

Preparation of total RNA

RNAs were extracted according to the FastRNA Pro protocol (Qbiogene), with slight modifications : three phenol/chloroform extraction in presence of 300 µL Tris HCl pH 7.5 100 mM were performed rather than using the Fast Pro Solution and chloroform extraction ; Fast prep was used twice for 45 seconds at speed 6.0 ; Sodium Acetate 0,3 M was added for RNA precipitation.

RNA-Seq library preparation and analysis

RNA-seq was performed as previously described[1]. Briefly, RNA was extracted as described above and was DNase treated and chemically fragmented. Strand specific RNA-seq libraries were prepared using the NEBNext® Ultra™ Directional RNA Library Prep Kit (NEB, E7420). Sequencing was performed using the Illumina NextSeq 500 and the data was deposited in the European Nucleotide Database (ENA) under accession no. PRJEB25942. Sequencing reads were mapped to the NC_003210 *Listeria monocytogenes* EGD-e RefSeq genome using NovoAlign (Novocraft) V3.02.02 with default parameters, discarding reads that were non-uniquely mapped. RNA-seq coverage shown in Figure S9A (*ErmC* induction) were reanalyzed using data from[1].

qRT–PCR.

cDNA were prepared as previously described[4]. Briefly, each sample was treated with DNase I (Turbo Q22 DNA-free kit, Ambion) and reverse-transcribed with Quantiscript Reverse Transcriptase (QuantiTect Reverse Transcription kit, Qiagen). qRT–PCR reactions were carried out and quantified with SYBR Green master mix on a C1000 Touch CFX384 machine (Biorad). Gene expression levels of genes were normalized to the *L. monocytogenes gyrA* gene, and the fold change was calculated using the delta-delta CT method. All samples were evaluated in triplicate and in at least three independent experiments. For statistics, we used an ordinary one-way or two-way Anova test (see legends) on ΔCt values, using biological replicates as pairing factors (* $p<0.05$; ** $p<0.01$, ns = non significant).

Western Blot

Bacteria were lysed in lysis buffer 150 mM KCl, 1 mM DTT, 25 mM Tris pH 7.4, Complete protease inhibitor cocktail (Roche) as described for preparation of total RNA. Concentration was estimated using the BCA protein assay kit (Thermo) or Bradford reagent (Bio-Rad). Proteins were loaded on 10% TGX stain-free gels (Biorad). Subsequent Western Blot experiment was performed as described in Mellin et al., 2014 [7], briefly after transfer on nitrocellulose membrane, a mouse anti-flag antibody (Sigma, F7425) (dilution 1:1000) was used, followed by HRP-antimouse antibody (AbCys, 1:4000 dilution), and signal was revealed using ECL Prime according to the manufacturer's

instructions and subsequently visualized on the Chemidoc touch imaging system. After membrane stripping using Restore PLUS Western Blot Stripping Buffer (Thermo 46430) according to the manufacturer's instructions, EF-Tu antibody (rabbit) was used as described in [8] (1:10000), followed by HRP-antirabbit antibody (AbCys, 1:4000 dilution). The experiments were reproduced twice independently.

Minimum inhibitory concentration

Minimum inhibitory concentration assays were performed as previously described[1]. Briefly, 6-8 colonies were resuspended in BHI at OD_{600}=0.001 in 96-well plates, and incubated in presence of increasing concentrations of antibiotics for 48h at 37°C without shaking and the MIC was determined as the lowest concentration to fully inhibit growth. The experiment was reproduced at least three time independently.

The highest concentration used were : 64 μg/ml for lincomycin, 1.44 μg/ml for erythromycin, tetracycline 4 μg/ml, chloramphenicol 32 μg/ml. The dilution was performed using a 2-fold increment.

Polysome profiles analysis

Listeria monocytogenes was grown overnight in Brain Heart Infusion (BHI) medium (Difco) at 37°C while shaking at 200 rpm. Overnight cultures were diluted 1/100 in fresh BHI and grown at 37°C. For ribosome splitting, erythromycin (0.18 μg/ml) was added or not (untreated condition) at OD_{600} = 0.6 and the growth was pursued for 1h. Chloramphenicol was quickly added to every cultures (2 min at 5 mM final concentration) in order to stabilize polysomes. The bacteria were pelleted by centrifugation (2 min at 28000 g), washed in 1mM Cam, and flash-frozen in liquid nitrogen. Pellets were resuspended in lysis buffer (20 mM Tris HCl pH 7.5, 100 mM NH_4Cl, 10 mM $MgCl_2$, 0.1% Nonidet p-40, 0.4% Triton x100, 1 mM Chloramphenicol) and cells were disrupted in the Fast-Prep apparatus, using 2 steps of 45 seconds shaking at 6.0 m/s, with a pause of 1 min at 4°C. Equal amount (15-35K) OD_{260} of cell lysates were loaded on sucrose gradient (5-50%) and separated by ultra-centrifugation (37K during 2h46). Samples were collected from top (0mm) to bottom (80mm) of the tube at a speed of 0.12 mm/sec using the Biocomp instrument, and absorbance was read at 260 nm. Raw data were exported,

the zero was set according to the inflexion point, and values were normalized according to the area under the curve. The percentage of 70S over total ribosomal fraction was determined by the area under the 70S peak divided by total area under the curve. Experiments were reproduced 3-5 time independently.

Computational analysis

Data

We analyzed 9078 complete genomes retrieved from NCBI RefSeq (ftp://ftp.ncbi.nih.gov/genomes/, last accessed in March 2018) representing 3226 species of Prokaryotes.

Identification of homologs

We took the two genes (*lmo0762* and *lmo1296*) and analyzed their composition in protein domains using PFAM (last accessed 20 September 2018, https://pfam.xfam.org/). We realized that both proteins had three well conserved domains (GTP-bdg_M, GTP-bdg_N, MMR_HSR). We used these domains to search the database of genomes using hmmsearch (from the package HMMER 3.1b2) [9]. We collected all proteins with hits better than the threshold suggested by PFAM (--cut_ga option). This resulted in 189954 hits, of which 172890 for MMR_HSR, 8527 for GTP-bdg_M and 8537 for GTP-bdg_N. A total of 8527 proteins had all three domains, showing that GTP-bdg_M and GTP-bdg_N almost always co-occur, and when they do there is always also a MMR_HSR domain. This suggests that the domain conservation of the protein is very high. To confirm these results, we also did a search for the protein using blastp v2.2.19+ (using a threshold $e<10^{-5}$), and fetched 8537 homologs in the genome database, of which 8527 had the three abovementioned PFAM domains. We thus used the 8527 proteins in all the following analyses.

Comparative genomics

We took all genomes from the database and separated them in species. Within each species, we initially filtered genomes that were either too divergent to be in the species or

very similar to existing genomes (redundant). For this, we computed genetic distances between all genomes within a species using Mash v2.0 (default parameters,[10]). The first sequenced genome of a species was defined as the reference genome of the species. If a genome was more than 6% divergent to the reference genome, we removed it from further analyses, since at this level of divergence the strain is probably not from the same species. Then we proceeded in the chronological list of complete genomes and added to the set of genomes to study all genomes with distances higher than 0.0001 to all the genomes already introduced in the set. This allows to remove redundant genomes resulting from multiple sequencing efforts of a similar strain. After this process, we obtained a list of 163 species for which we had at least five non-redundant complete genomes.

We computed the pan-genome of each species using mmseqs2 v 7319ccbb3ec80cbe6fa9d1b4c9527abea0e11e5c[11]. For this we computed pairwise similarities between all proteins and clustered them (--min-seq-id 0.80). We then computed the persistence of a given gene family in the pan-genome as the fraction of all genomes in the species containing at least one gene from the same gene family (Fig. S10 and Dataset S3).

Phylogenetic trees

The dataset of 8527 proteins is too large to make efficient phylogenetic analyses. Furthermore, it includes many proteins that are very similar because they correspond to homologs of species for which many genomes are available in the database (e.g., 99 for *Listeria monocytogenes*). We therefore started by reducing the redundancy of the dataset. We used mmseqs2 to cluster the proteins in clusters of 80% identity (dataset_80, 1216 clusters) using options considered to lead to very accurate searches (-s 7.5 --min-seq-id 0.80) and 60% identity (dataset_60 contains 347 clusters obtained with options -s 7.5 --min-seq-id 0.60). We picked the representative of each cluster in each case.

We identified the genomes encoding at least two copies of the protein. For each species, we kept the two copies for one of the strains (the reference strain if it contained the copy, otherwise a random strain). We then added all these pairs of proteins to the two datasets above (dataset_80 and dataset_60) to ensure that the phylogenetic reconstruction

contained the two copies of each pair of duplicates. This resulted in datasets with 1381 (dataset_80) and 594 (dataset_60) protein sequences.

We made a phylogenetic reconstruction for each of the two datasets in a similar way. First, we made multiple alignments with with MAFFT v7.407 (with sensitive option --maxiterate 1000) [12]. We then collected the informative sites in multiple alignment using trimAl v1.2rev59 (option -gappyout) [13]. These were used to make a phylogenetic reconstruction with IQtree v1.6.7 (options -m TEST -bb 1000). The best-fit models were LG+I+G4 (dataset_60) and LG+F+I+G4 (dataset_80). The dataset_60 reconstruction was used to analyzed the data because it had more solid ultra-fast bootstrap results and fewer taxa. Yet, the key results were common between the two reconstructions.

Analysis of conserved neighborhoods

We analyzed the neighborhood of the genes in the clades of *lmo0762* and *lmo1296* in the phylogenetic tree computed with the dataset_60. We recovered the genes of the clades, corresponding respectively to 100 (*lmo0762*) and 64 (*lmo1296*) genes. For each gene, we recovered the 10 genes on each side of the gene in the replicon. We then clustered all these genes by sequence similarity (minimal 40% identity) for each clade separately using mmseqs2 (-s 7.5 --min-seq-id 0.4). We analyzed all families with more than 10 genes.

1. Dar D, Shamir M, Mellin JR, et al (2016) Term-seq reveals abundant ribo-regulation of antibiotics resistance in bacteria. Science (80-) 352:1–12 . doi: 10.1126/science.aad9822

2. Arnaud M, Chastanet A, De M (2004) New Vector for Efficient Allelic Replacement in Naturally Gram-Positive Bacteria New Vector for Efficient Allelic Replacement in Naturally Gram-Positive Bacteria †. Appl Enviromental Microbiol 70:6887–6891 . doi: 10.1128/AEM.70.11.6887

3. Mellin JR, Tiensuu T, Bécavin C, et al (2013) A riboswitch-regulated antisense RNA in Listeria monocytogenes. Proc Natl Acad Sci 110:13132–13137 . doi: 10.1073/pnas.1304795110

4. Impens F, Rolhion N, Radoshevich L, et al (2017) N-terminomics identifies Prli42 as a membrane miniprotein conserved in Firmicutes and critical for stressosome activation in Listeria monocytogenes. Nat Microbiol 17005:1–12 . doi:

10.1038/nmicrobiol.2017.5

5. Balestrino D, Anne Hamon M, Dortet L, et al (2010) Single-cell techniques using chromosomally tagged fluorescent bacteria to study Listeria monocytogenes infection processes. Appl Environ Microbiol 76:3625–3636 . doi: 10.1128/AEM.02612-09

6. Lauer P, Yin M, Chow N, et al (2002) Construction , Characterization , and Use of Two Listeria monocytogenes Site-Specific Phage Integration Vectors Construction , Characterization , and Use of Two Listeria monocytogenes Site-Specific Phage Integration Vectors. J Bacteriol 184:4177–4186 . doi: 10.1128/JB.184.15.4177

7. Mellin JR, Koutero M, Dar D, et al (2014) Sequestration of a two-component response regulator by a riboswitch-regulated noncoding RNA. Science 345:940–943 . doi: 10.1126/science.1255083

8. Archambaud C, Gouin E, Pizarro-Cerda J, et al (2005) Translation elongation factor EF-Tu is a target for Stp, a serine-threonine phosphatase involved in virulence of Listeria monocytogenes. Mol Microbiol 56:383–396 . doi: 10.1111/j.1365-2958.2005.04551.x

9. Eddy SR (2011) Accelerated profile HMM searches. PLoS Comput Biol 7:1–16 . doi: 10.1371/journal.pcbi.1002195

10. Ondov BD, Treangen TJ, Melsted P, et al (2016) Mash: Fast genome and metagenome distance estimation using MinHash. Genome Biol 17:1–14 . doi: 10.1186/s13059-016-0997-x

11. Steinegger M, Söding J (2017) MMseqs2 enables sensitive protein sequence searching for the analysis of massive data sets. Nat Biotechnol 35:1026–1028 . doi: 10.1038/nbt.3988

12. Katoh K, Standley DM (2013) MAFFT multiple sequence alignment software version 7: Improvements in performance and usability. Mol Biol Evol 30:772–780 . doi: 10.1093/molbev/mst010

13. Capella-Gutiérrez S, Silla-Martínez JM, Gabaldón T (2009) trimAl: A tool for automated alignment trimming in large-scale phylogenetic analyses. Bioinformatics 25:1972–1973 . doi: 10.1093/bioinformatics/btp348

14. Antunes LCS, Poppleton D, Klingl A, et al (2016) Phylogenomic analysis supports the ancestral presence of LPS-outer membranes in the firmicutes. Elife 5:1–21 . doi: 10.7554/eLife.14589

15. Sharkey L, Edwards T, O'Neill A (2016) ABC-F Proteins Mediate Antibiotic Resistance through Ribosomal Protection. MBio 7:1–10 . doi: 10.1128/mBio.01975-15.Editor

16. Sharkey LKR, O'Neill AJ (2018) Antibiotic Resistance ABC-F Proteins: Bringing Target Protection into the Limelight. ACS Infect Dis 4:239–246 . doi: 10.1021/acsinfecdis.7b00251

17. Boël G, Smith PC, Ning W, et al (2014) The ABC-F protein EttA gates ribosome entry into the translation elongation cycle. Nat Struct Mol Biol 21:143–151 . doi: 10.1038/nsmb.2740

18. Ramu H, Mankin A, Vazquez-Laslop N (2009) Programmed drug-dependent ribosome stalling: MicroReview. Mol Microbiol 71:811–824 . doi: 10.1111/j.1365-2958.2008.06576.x

19. Sothiselvam S, Liu B, Han W, et al (2014) Macrolide antibiotics allosterically predispose the ribosome for translation arrest. Proc Natl Acad Sci 111:9804–9809 . doi: 10.1073/pnas.1403586111

20. Plante I, Centrón D, Roy PH (2003) An integron cassette encoding erythromycin esterase, ere(A), from Providencia stuartii. J Antimicrob Chemother 51:787–790 . doi: 10.1093/jac/dkg169

21. Bozdogan B, Galopin S, Leclercq R (2004) Characterization of a New erm-Related Macrolide Resistance Gene Present in Probiotic Strains of Bacillus clausii. Appl Environ Microbiol 70:280–284 . doi: 10.1128/AEM.70.1.280

22. Gryczan T, Israeli-Reches M, Del Bue M, Dubnau D (1984) DNA sequence and regulation of ermD, a macrolide-lincosamide-streptogramin B resistance element from Bacillus licheniformis. MGG Mol Gen Genet 194:349–356 . doi: 10.1007/BF00425543

23. Berryman DI, Lyristis M, Rood JI (1994) Cloning and sequence analysis of ermQ, the predominant macrolide-lincosamide-streptogramin B resistance gene in Clostridium perfringens. Antimicrob Agents Chemother 38:1041–6

24. Serwold-davis TM, Neal BG (1988) Identification of a methylase gene for erythromycin resistance within the sequence of a spontaneously deleting fragment of Corynebacterium diphtheriae plasmid pNG2. FEMS Microbiol Lett 56:7–14

25. Gay K, Stephens DS (2001) Structure and dissemination of a chromosomal insertion element encoding macrolide efflux in Streptococcus pneumoniae. J Infect Dis 184:56–65 . doi: JID010038 [pii] 10.1086/321001

26. Matsuoka M, Jánosi L, Endou K, Nakajima Y (1999) Cloning and sequences of inducible and constitutive macrolide resistance genes in <open_i>Staphylococcus aureus<close_i> that correspond to an ABC transporter. FEMS Microbiol Lett 181:91–100 . doi: 10.1016/S0378-1097(99)00518-2

27. Ross JI, Eady EA, Cove JH, et al (1990) Inducible erythromycin resistance in staphlyococci is encoded by a member of the ATP-binding transport super-gene

family. Mol Microbiol 4:1207–1214 . doi: 10.1111/j.1365-2958.1990.tb00696.x

28. Matsuoka M, Endou K, Kobayashi H, et al (1998) A plasmid that encodes three genes for resistance to macrolide antibiotics in *Staphylococcus aureus*. FEMS Microbiol Lett 167:221–7 . doi: 10.1111/j.1574-6968.1998.tb13232.x

29. Singh K V., Malathum K, Murray BE (2001) Disruption of an Enterococcus faecium species-specific gene, a homologue of acquired macrolide resistance genes of staphylococci, is associated with an increase in macrolide susceptibility. Antimicrob Agents Chemother 45:263–266 . doi: 10.1128/AAC.45.1.263-266.2001

30. Reynolds E, Ross JI, Cove JH (2003) Msr(A) and related macrolide/streptogramin resistance determinants: Incomplete transporters? Int J Antimicrob Agents 22:228–236 . doi: 10.1016/S0924-8579(03)00218-8

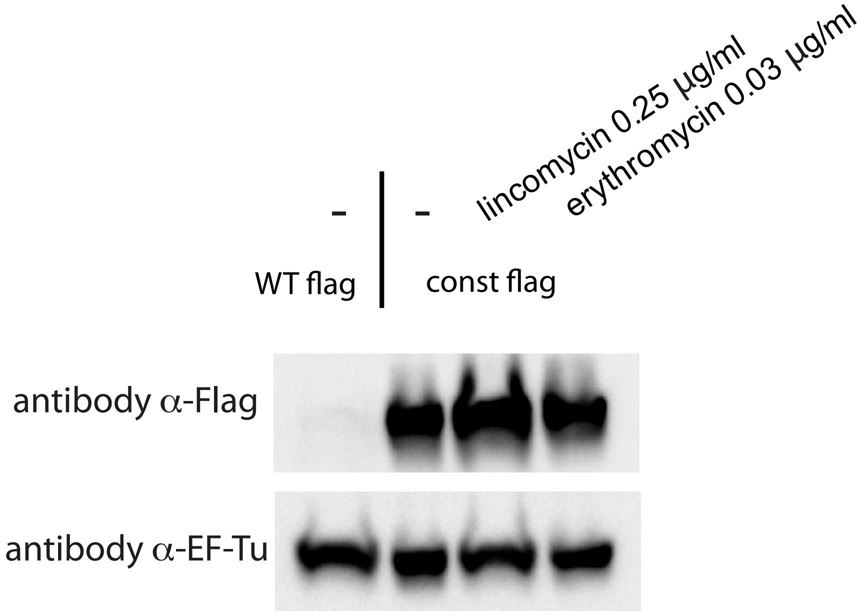

Figure S1 : the mutant *const-flag* constitutively express Lmo0762 protein

Bacteria EGDe carrying the mutation in the regulatory region that we name "const" (for constitutive expression) in which in inserted a flag tag at the C-terminus of Lmo0762 (const-flag) were grown for 1h in presence or in absence of antibiotics and total protein were extracted. Western blot analysis using anti-flag antibody revealed a constitutive induction of Lmo0762-flag in this strain. EF-Tu was used as a loading control

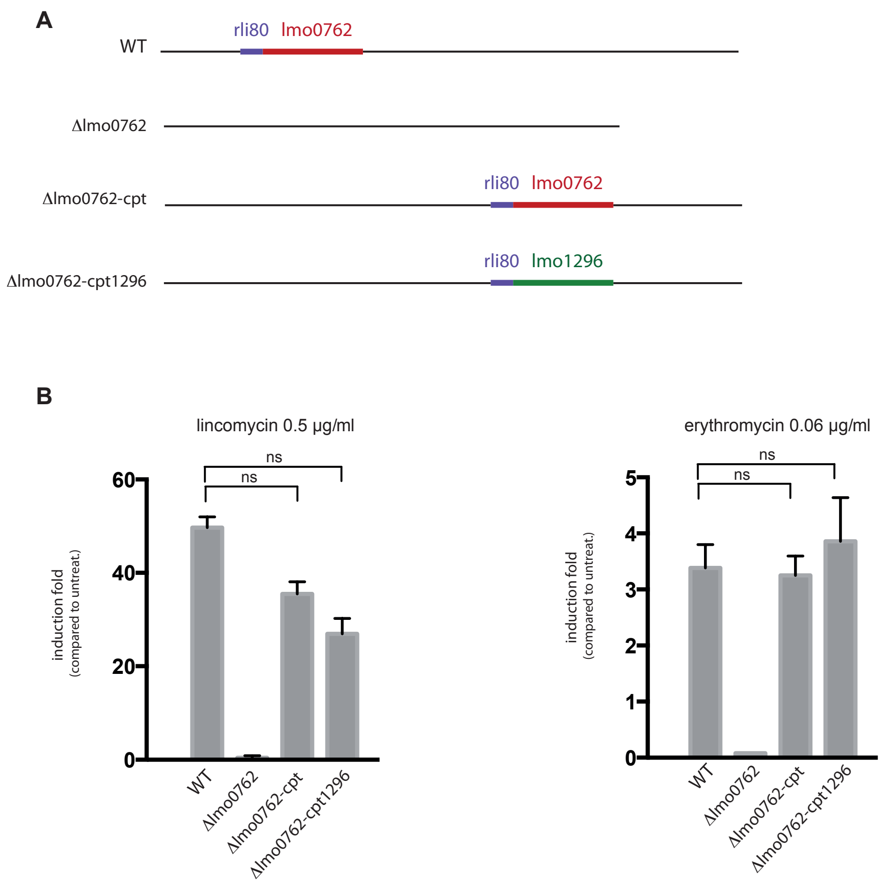

Figure S2 : control of rli80-regulated gene induction in different strains

A. Schematic representation of the different strains' genome used in this assay. B. qRT-PCR assay shows that in the two complemented strains, the gene located downstream rli80 is induced by antibiotics in a similar manner. The qPCR primers used in this assay amplify the rli80 region only in its long form, and thus can be used to detect induction of all rli80-regulated genes shown in 1A. Statistical analysis was performed using a two-way anova on ΔCt values, using biological replicates as pairing factors ($p<0.05$), ns = non significant.

	ery	lin	tetr	cam
WT	0.18	16	1	8
Δ0762	0.09	16	1	8
Δ0762 cpt	0.18	16	1	
Δ0762 cpt 1296	0.09	16	1	
Δ0919	0.18	2	1	8
Δ0919- Δ0762	0.09	0.5	1	8
Δ0919- Δ0762 cpt	0.18	2	2	
Δ0919-const	0.72	4	1	8
Δ1296	0.18	16	1	8
Δ0762- Δ1296	0.09	16	1	8
const	0.72	8	2	16
anti	0.18	16	1	8
ATG	0.09	16	1	8

Figure S3 : MIC experiment of various strains in presence of different antibiotics
Legend similar to figure 1C. ery : erythromycin; lin : lincomycin; tetr : tetracycline; cam : chloramphenicol. MIC values after 48h incubation are also indicated within each box (μg/ml). The crossed boxes correspond to strains that carry Cam^R gene due to pAD insertion, and were not considered in this experiment.

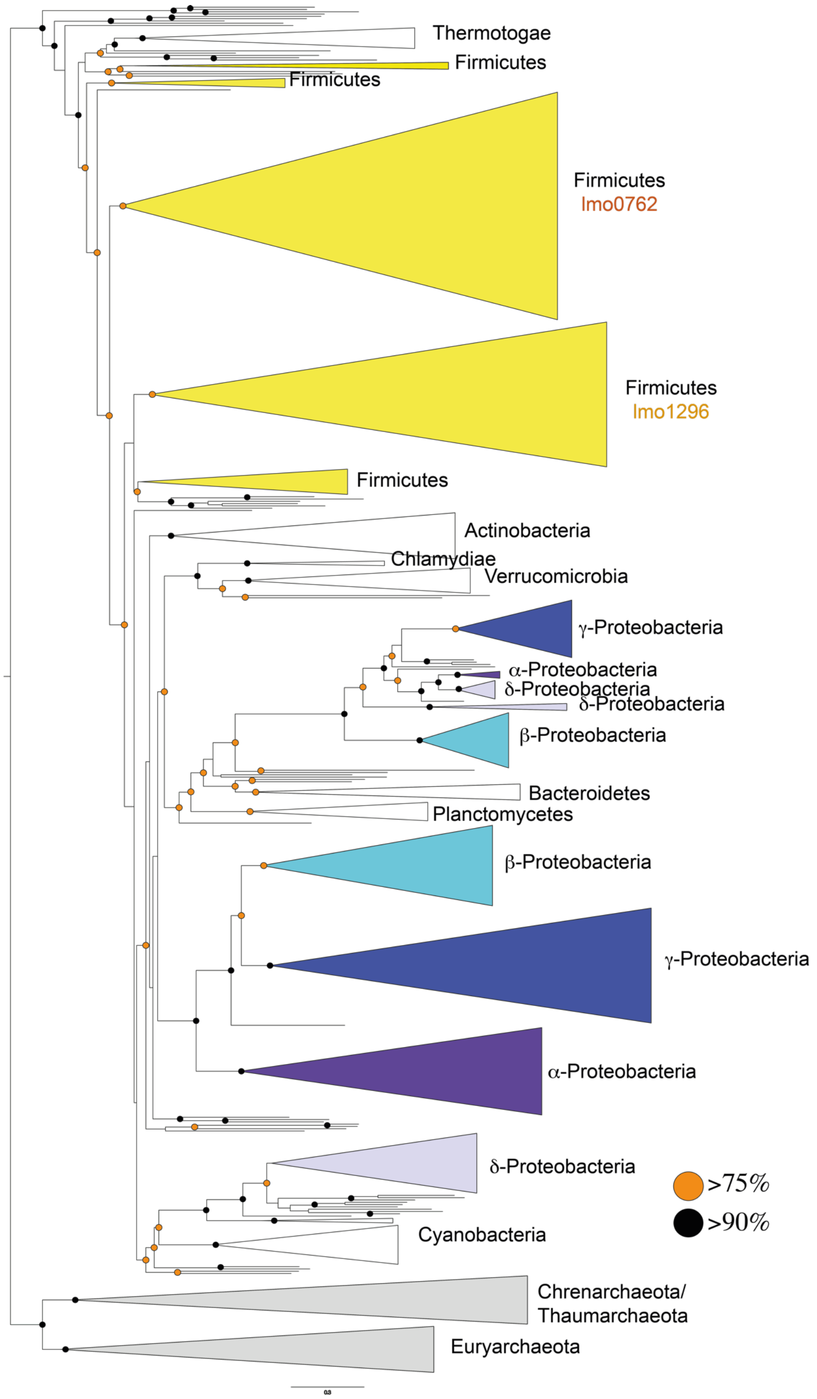

Figure S4: Phylogenetic tree of HflX proteins (dataset_60). The circles represent ultra-fast bootstrap support (out of 1000 experiments). The nodes with low support do not have a circle. Clades with extensive duplications are colored. Archaea, where duplications are within each major clade, are in grey. The labels in orange indicate the two clades where lmo0762 and lmo1286 are in the tree. The full tree file that can be visualized in a tree visualizer is given as supplementary dataset S6.

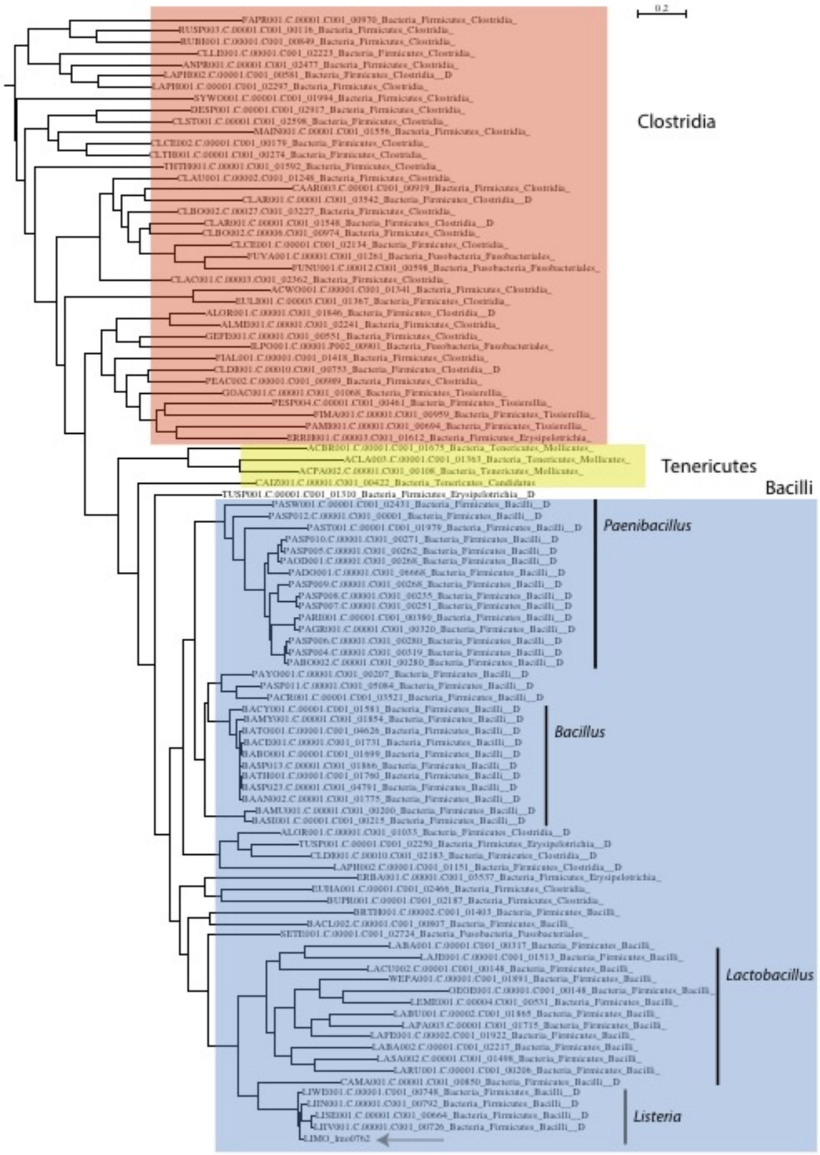

Figure S5: Detail of the phylogenetic tree of *hflX*, focused on the clade containing *lmo0762*. The tree coarsely recapitulates the tree of the firmicutes (e.g., see[14]), with initial splits concerning the Clostridia, and, within Bacilli, Listeria appearing as the sister clade of Lactobacillus. The arrow indicates the position in the tree of *lmo0762*. The explanation of the codes of the names of taxa are indicated in supplementary dataset S4 and S5.

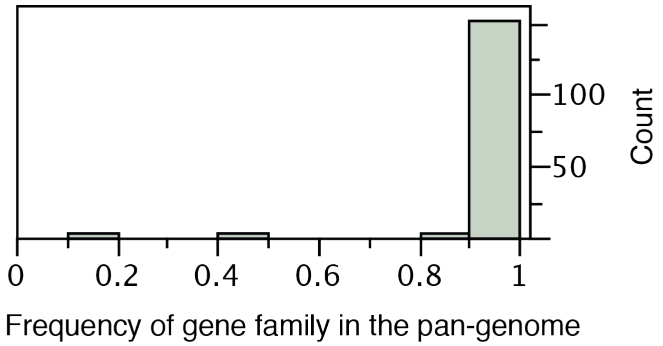

Figure S6: Frequency of gene family in the pan-genome. The gene *hflX* was in most cases either absent of the pan-genome (19 species, mostly in Campylobacterales and Mollicutes) or present in more than 90% of the strains of the species. The graph represents the frequency of gene families of *hflX* in the pan-genomes of 163 species for species with at least one gene in one genome of the species. Duplicated *hflX* genes were usually present in different pan-genome families, because of their low sequence similarity, and both copies were present in more than 90% of the strains.

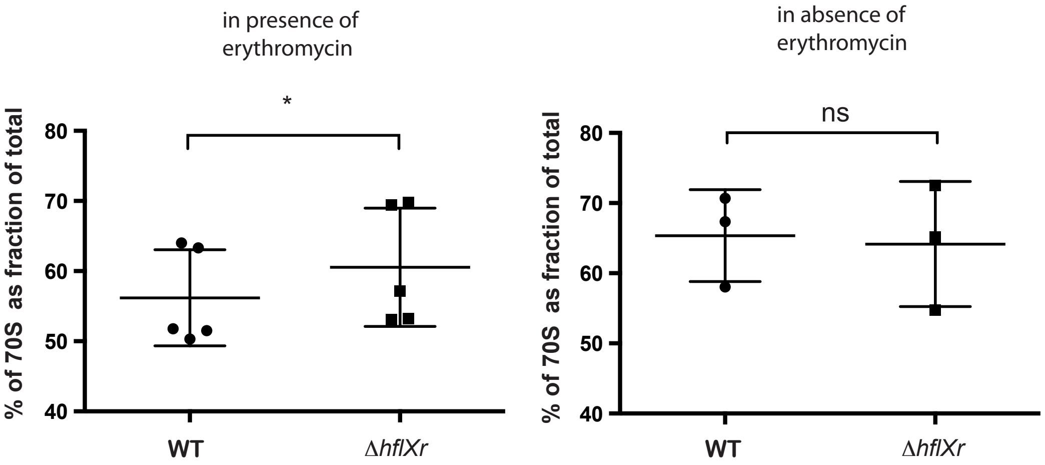

Figure S7. Comparison of 70S amount in comparison to total ribosomal fraction between WT and Δ*hflXr* strains. The values were obtained by calculating the percent of 70S as fraction of total ribosomal content in WT and Δ*hflXr* strains. The data are presented in mean ± SD. A significant difference of 70S fraction between WT and Δ*hflXr* strains was observed in presence of erythromycin, but not in absence of the antibiotic. Statistical analysis was performed using paired t-tests on the percent of 70S fraction in WT and Δ*hflXr* strains using replicates as pairing factor (in presence of ery : t=3.691 df=4, p-value =0.021 n=5 ; in absence of ery t=0.765 df=2, p-value=0.5242 n=3).

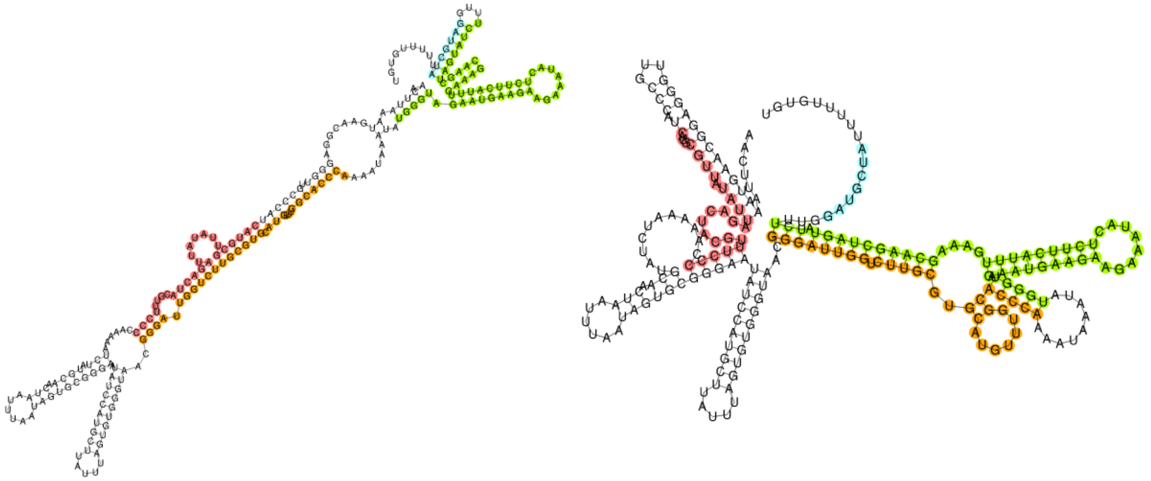

Figure S8. rli80 RNA can adopt terminator and anti-terminator structures. RNA sequence of rli80 was analysed using PASIFIC software, which indicated that rli80 can adopt two mutually exclusive structures, as a terminator and as a anti-terminator. Color code was drawn as in fig. 4A.

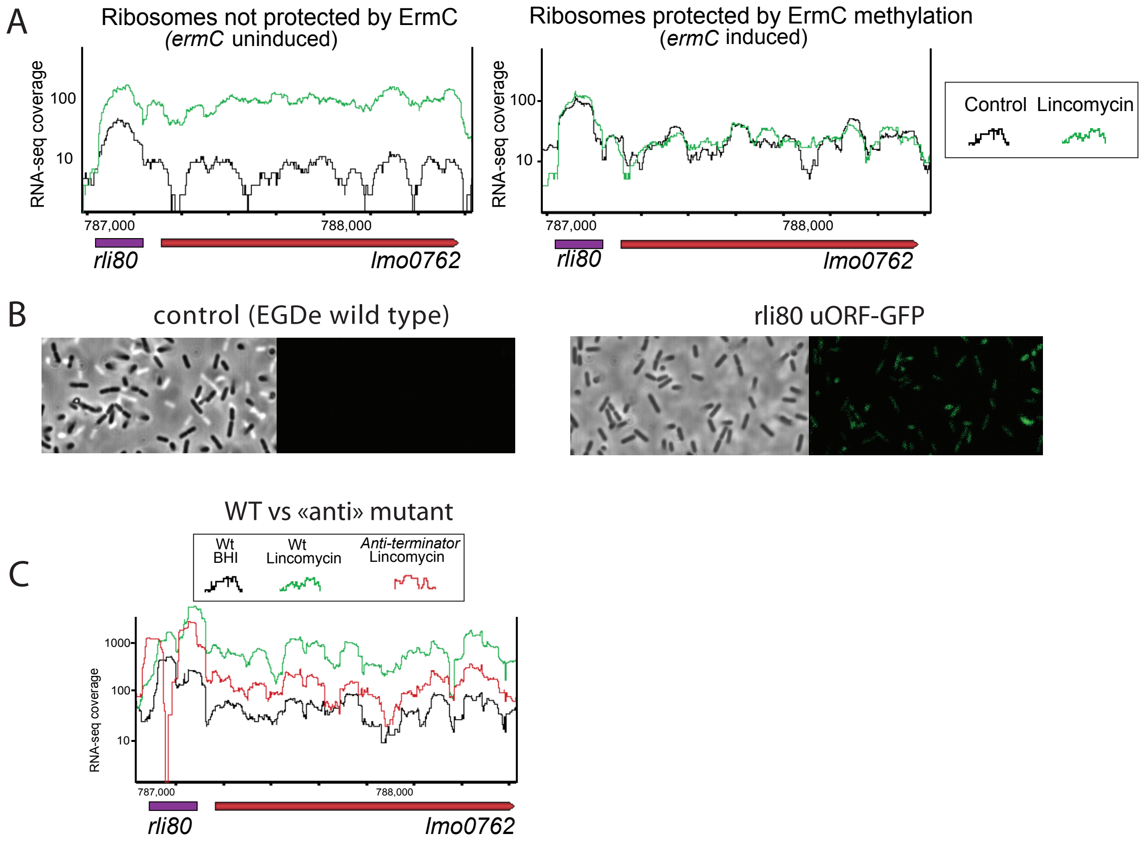

Figure S9. Attenuation of transcription depends on ribosome stalling

(A) Wild-type bacteria in which *ermC* expression was induced or not were grown until exponentional phase in BHI, and bacteria were collected before (black RNA-seq coverage), or after 15 min exposure to sub-inhibitory concentration (0.25 μg/ml) of lincomycin (green) and RNA was extracted and sequenced. The RNA-seq profile of the *rli80-lmo0762* locus is presented here. RNA-Seq coverage were reanalysed using data from [1]. (B) The *L. monocytogenes lmo0762* ribo-regulator (rli80) was modified by a chromosomal in-frame fusion of a GFP reporter protein that lacks the initiation codon, to the 14 aa ORF. Left and right panels show the phase contrast and fluorescence images, respectively, and demonstrate that the ORF is translated *in-vivo*. (C) RNA-Seq profiles of WT and mutant bacteria, obtained as in Fig. 1A.

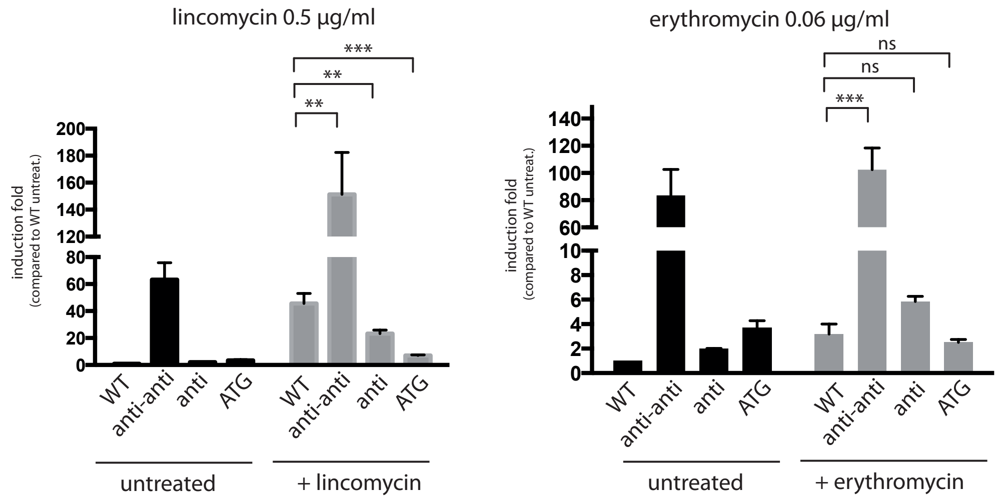

Figure S10. Mutations in rli80 leader region induces or represses lmo0762 induction, according to the attenuation model.

RNA was extracted from wild-type bacteria grown in BHI, before and after 15 min exposure to lincomycin (0.5 μg/ml) or erythromycin (0.06 μg/ml). Induction of *lmo0762* was calculated by comparison with the mRNA level in WT strain before the addition of the antibiotic. We used a two-way anova on ΔCt values for statistics, using biological replicates as pairing factors ($p<0.05$). Data are represented as mean ± s.e.m.

* p-value < 0.05, ** p-value < 0.01, *** p-value <0.005, ns = non significant

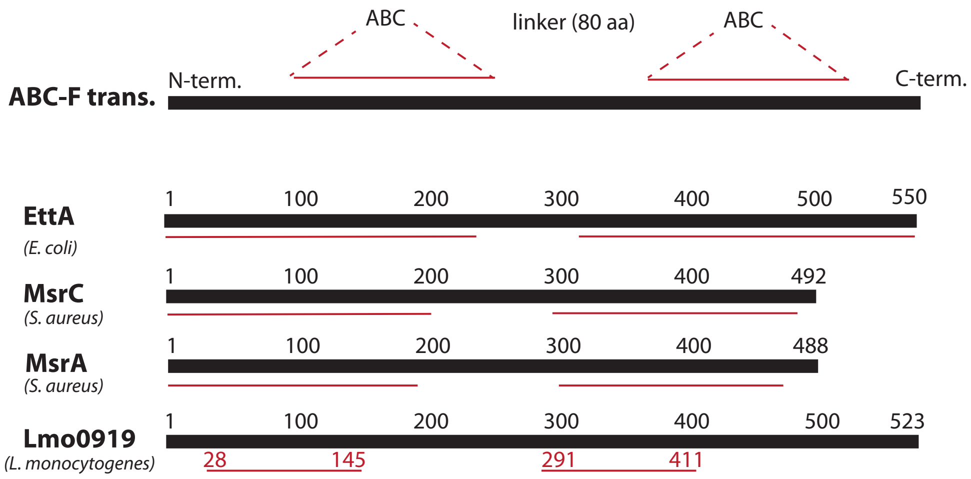

Figure S11 Lmo0919 is an ABC-F transporter. Comparison of the conserved domains of Lmo0919 and other bacterial ABC-F proteins, using the NCBI conservation domain algorithm. The presence of transmembrane domains was analysed using the TMHMM Server v. 2.0. Lmo0919 is predicted to contain two ATP-binding cassettes (ABC, red) separated by a linker region, and no transmembrane domains. These features are typical of members of the ABC-F subfamily of ABC transporters[15–17]. Protein accession numbers: AB013298 (MsrA), AY004350 (MsrC), P0A9W3 (EttA), NP_464445 (Lmo0919).

Table S1. BlastP analysis between HflX proteins of *Listeria* and other bacteria. We used the BLASTp algorithm to determine the percentage of similarity (bottom left) and identity (top right) of HflX from various species. The accession number used were *Bacillus cereus* HflX WP_098490556, *Staphylococcus aureus* HflX WP_031764191, *Escherichia coli* HflX WP_001608794, *Listeria monocytogenes* Lmo0762 WP_075491923, *Listeria monocytogenes* Lmo1296 WP_070753220.

species	*Bacillus cereus* HflX	*Staphylococcus aureus* HflX	*Escherichia coli* HflX	*Listeria monocytogenes* Lmo0762	*Listeria monocytogenes* Lmo1296
Bacillus cereus HflX	100	41	39	38	38
Staphylococcus aureus HflX	63	100	44	48	48
Escherichia coli HflX	61	62	100	40	37
Listeria monocytogenes Lmo0762	58	68	59	100	51
Listeria monocytogenes Lmo1296	58	67	56	68	100

% ident

% similarity

Table S2. The GTP binding domains and 50S binding domains are conserved between HflX proteins of *Listeria* and other bacteria. We used PFAM algorithm to identify the domains present in the different proteins.

		Lmo1296		Lmo0762		*S.aureus* HflX		*E.coli* HflX	
	aa from N-term	start	stop	start	stop	start	stop	start	stop
GTP-bdg_N	GTP-binding GTPase N-terminal	22	110	18	106	32	120	24	112
GTP-bdg_M	GTP-binding GTPase Middle Region	112	190	108	188	122	200	114	192
MMR_HSR1	50S ribosome-binding GTPase	197	316	195	321	207	326	199	318

Table S3. RLR motif is found upstream of macrolide resistance genes

Many macrolide resistance genes are located downstream a small ORF which contains the RLR motif. We selected few examples of these genes (list modified from [18]), a more complete list is available in [19]

Name of the regulated gene	Sequence of the leader peptide	Mode of action of the regulated gene	Species	References
ereA	MLRSRAVALKQSYAL	erythromycin modification (esterase)	*Providencia stuartii*	[20]
erm34	MHFIRLRFLVLNK	rRNA methylase	*Bacillus clausii*	[21–24]
ermD	MTHSMRLRFPTLNQ		*Bacillus licheniformis*	
ermQ	MIMNGGIASIRLRR		*Clostridium perfringens*	
ermX	MLISGTAFLRLRTNR		*Corynebacterium diphtheriae*	
mefE	MTASMRLR	Transport (efflux)	*Streptococcus pneumoniae*	[25, 26]
msrA	MTASMRLK	ABC-F transporter (antibiotic displacement)	*Staphylococcus aureus*	[27–30]
msrSA	MTASMRLK		*Staphylococcus aureus*	
msrC	MTASMKLRFELLNNN		*Enterococcus faecium*	
hflXr	MRYIRLRFPKNLCN	Ribosome splitting	*Listeria monocytogenes*	This study

Table S4. Strains and plasmids used in this study

Strains	Characteristics	Collection no.	Source or reference
EGD-e	*Listeria monocytogenes* EGD-e WT strain	BUG1600	Mackanes et al 1964
Δlmo0762	*Listeria monocytogenes* EGD-e rli80-*lmo0762* deletion mutant : positions 787045 to 787267 (rli80) and 787305 to 788553 (*lmo0762* ORF) deleted	BUG4131	this study
Δlmo0762-cpt	*Listeria monocytogenes* rli80-*lmo0762* deletion mutant (BUG4131) complemented using pAD-rli80-*lmo0762*	BUG4132	this study
Δlmo0762-cpt1296	*Listeria monocytogenes* rli80-*lmo0762* deletion mutant (BUG4131) complemented using pAD-rli80-*lmo1296*	BUG4280	this study
Δlmo1296	*Listeria monocytogenes lmo1296* deletion mutant	BUG4247	this study
Δlmo1296 Δlmo0762	*Listeria monocytogenes lmo1296* deletion mutant BUG 4247 in which rli80-*lmo0762* region has been deleted	BUG4296	this study
Δlmo0919	*Listeria monocytogenes lmo0919* deletion mutant	BUG3846	Dar er al 2016
Δlmo0919Δlmo0762	*Listeria monocytogenes* rli80-*lmo0762* deletion mutant derived from BUG3846	BUG4171	this study
Δlmo0919Δlmo0762-cpt	*Listeria monocytogenes lmo0919*-rli80-*lmo0762* deletion mutant (BUG4171) complemented using pAD-rli80-*lmo0762*	BUG4241	this study
Δlmo0919const	*Listeria monocytogenes lmo0919* deletion mutant in which the mutation antianti has been introduced leading to constitutive expression of *lmo0762*	BUG4281	this study
const	*Listeria monocytogenes* EGD-e where regulatory region anti-anti-terminator of rli80 has been removed (-7 to -4 mutated in tttt and +7 to +29 has been deleted, A of the initiator ATG being the +1) leading to constitutive expression of *lmo0762*	BUG4118	this study
anti	*Listeria monocytogenes* EGD-e where regulatory region of rli80 anti-terminator has been removed (+57 to +103 and +116 to +120 have been deleted, A of the initiator ATG being the +1)	BUG4120	this study
rli80 uORF ATG	*Listeria monocytogenes* EGD-e where the initiator ATG of the small ORF encoded in rli80 has been mutated in ACG	BUG4125	this study
rli80 uORF-GFP	*Listeria monocytogenes* EGD-e where a GFP gene has been introduced at the C-terminus of the protein encoded by the small ORF of rli80	BUG4242	this study
Lmo0762-flag	*Listeria monocytogenes* EGD-e WT, where two FLAG were introduced at the C-terminus of *lmo0762*	BUG4175	this study
const-flag	*Listeria monocytogenes* EGD-e antianti/const (BUG4118), where two FLAG were introduced at the C-terminus of *lmo0762*	BUG4282	this study

Plasmids	Characteristics	Collection no.	Source or reference
pMAD	shuttle vector used for cretaing plasmid for mutagenesis	BUG1957	(Arnaud et al., 2004)

pMAD-antiantiterm1-rli80	plasmid used to create the const mutant	BUG4109	this study
pMAD-antiterm-rli80	plasmid used to create the anti-term mutant	BUG4114	this study
pMAD-atg-rli80	plasmid used to create the rli80 uORF ATG mutant	BUG4105	this study
pMAD-Δlmo0762Δrli80	plasmid used to create the Δlmo0762 mutant	BUG4113	this study
pAD-rli80-lmo0762	integrative plasmid to create the Δlmo0762-cpt and Δlmo0919Δlmo0762-cpt strains	BUG4243	this study
pAD-rli80-lmo1296	integrative plasmid to create the Δlmo0762-cpt1296 strain	BUG4283	this study
pMAD-lmo1296	plasmid used to create the Δlmo1296 mutant	BUG4244	this study
pMAD-lmo0762-flag	plasmid used to create the Lmo0762-flag mutant	BUG4245	this study
pMAD-rli80 uORF-GFP	plasmid used to create the rli80 uORF-GFP mutant	BUG4246	this study
pAD-pActA-YFP	plasmid used to create the pAD-rli80-lmo0762 plasmid	BUG2794	Balestrino et al., 2010

**Primer**	**Sequence**
**Oligonucleotides used to amplify synthetic DNA**	
Del_lmo1296-A	CTGATCGAATTCATTCGCGGCATTTCGCG
Del_lmo1296-D	GAGTCAGGATCCTGGATTGATATTTTTACAAATATGATCATTTC
5' gblock uORF-GFP	ATCGCCGAATTCCTGTTCAAC
3' gblock uORF-GFP	GGCGATGTCGACTTCGC
**Oligonucleotides used to sequence the pMAD plasmids and mutants**	
5’ rli80	GAAAACTACAAATTTTTAACTGGAAGC
3’ middle 0762	TAGCAAAGAGCATGTCTTTCTC
3’ downstrm 0762	TTAGCTGTCGCATCGTCTAAAG
3-stopregion-lmo0726	CTCCTCACTAAATTTGCTGTATG
lmo1296 upstream	GAA GAA TGT CGC TTT AAA TC
lmo1296 downstream	TCG CTT GAT TAA ATT CAG C
pMAD-up	aag cga gaa gaa tca taa tgg gga agg
pMAD-down-v2	cat aat tat tcc ccc tag cta att ttc gt
**Oligonucleotides to create pAD-cpt plasmid**	

compl rli80_lmo0762 up	AGTGAACCCGGGCACCAGTTGCCAGACTCGCTG
compl rli80_lmo0762 down	AGTGAAGTCGACTTTGCTGTATGTTTCATTATAGC
**Sequencing insert in pAD-based plasmid**	
pPL2-Fw	TTCGACCCGGTCGTCGGTTC
pPL2-Rv	CTTAGACGTCATTAACCCTCAC
**Verification of pAD integration in the *Listeria* chromosome**	
NC16	GTCAAAACATACGCTCTTATC
PL95	ACATAATCAGTCCAAAGTAGATGC
**Oligonucleotides used to perform qRT-PCR**	
qPCR-lmo0762-for	GGAAGTGGTGGGCGAAATAC
qPCR-lmo0762-rev	ATAATCAAGCGAGCGTCCTG
qPCR-gyrA-for	TCGGCATGGAAGTACTGGAG
qPCR-gyrA-rev	ACACCCATACCACCACGATT
qPCR-lmo0919-for	AGCGTTCAAACCAAGCAAGT
qPCR-lmo0919-rev	CAGCAACTTCACTCGTTCCC
qPCR-lmo1296-for	CTGCGCTAGTGGAAATGCAT
qPCR-lmo1296-rev	TTCGATCAATAATCCGCGCC
qRTrpoB-F	GCGAACATGCAACGTCAAGCAGTA
qRTrpoB-R	ATGTTTGGCAGTTACAGCAGCACC
qRT-rli80-long-for	GGG TAG AAT GAA GAA GAA ATA CTC
qRT-rli80-long-rev	AGG ACC TCC TGA AAT AGA TTC
